# Elevated DYRK1A in Primary Tauopathies and Therapeutic Targeting with the Brain-Penetrant Inhibitor DYR533

**DOI:** 10.21203/rs.3.rs-10733156/v1

**Published:** 2026-08-25

**Authors:** Ramon Velazquez, Samantha Bartholomew, Wendy Winslow, Savannah Tallino, Christopher Foley, Yeng Shaw, Samantha Rokey, Jessica Judd, Thomas Beach, Geidy Serrano, Gerrit Wilms, Ragini Kushwahag, Aidan McMahon, Sean Ginn, Walter Becker, Christopher Hulme, Travis Dunckley

**Affiliations:** Arizona State University Biodesign Institute; Arizona State University Biodesign Institute; Banner Sun Health Research Institute; Banner Sun Health Research Institute

## Abstract

Neurodegenerative disorders are increasing in prevalence, yet disease-modifying therapies remain limited. Dual-specificity tyrosine-phosphorylation-regulated kinase 1A (DYRK1A) phosphorylates tau and regulates inflammatory signaling, making it a potential therapeutic target for neurodegenerative diseases. To investigate the relevance of DYRK1A to primary tauopathies, we first evaluated DYRK1A protein levels in the superior frontal gyrus of individuals with Pick’s disease, corticobasal degeneration, and progressive supranuclear palsy. DYRK1A protein expression was significantly elevated in individuals with primary tauopathies compared with healthy controls and positively correlated with Braak stage. Conversely, DYRK1A levels inversely correlated with last Mini-Mental State Examination (MMSE) scores and brain weight, linking elevated DYRK1A expression to disease severity. We next developed DYR533, a selective, orally bioavailable, brain-penetrant small-molecule DYRK1A inhibitor with an S(35) score of 1.4 nM based on a 403-target KINOMEscan assay. Mechanistically, DYR533 prevented the autophosphorylation of newly translated DYRK1A, rendering the kinase inactive and thereby inhibiting phosphorylation of downstream substrates. We next evaluated the therapeutic efficacy of DYR533 in the PS19 mouse model of primary tauopathy, assessing tau hyperphosphorylation, neuroinflammation, motor function, and spatial cognition. DYR533 reduced tau hyperphosphorylation at threonine 217, threonine 181, and serine 396, and attenuated the expression of neuroinflammatory cytokines and chemokines implicated in disease progression. In PS19 mice, DYR533 treatment produced modest improvements on behavior. Together, these findings establish an association between elevated DYRK1A and disease severity in human primary tauopathies and demonstrate that pharmacological inhibition of DYRK1A with DYR533 reduces pathological tau phosphorylation and neuroinflammatory signaling in vivo.

## 1 Background

The prevalence of neurodegenerative diseases increases annually, necessitating therapies to prevent or slow disease progression. Tauopathies comprise a heterogeneous class of neurodegenerative disorders characterized by the pathological accumulation, hyperphosphorylation, and propagation of tau, culminating in the formation of neurofibrillary tau tangles (NFTs). Tauopathies can be categorized as primary or secondary, depending on whether tau precedes or succeeds other proteinopathies, respectively. Examples of primary tauopathies include Frontotemporal dementia – tau (FTD-tau), corticobasal degeneration (CBD), progressive supranuclear palsey (PSP), and Pick’s disease, whereas Alzheimer’s disease (AD) is a secondary tauopathy since amyloid-β (Aβ) deposition precedes tau aggregation. Unlike the major therapeutic advances made in the treatment of Aβ pathology(1), therapeutic strategies aimed at mitigating tau pathogenesis have thus far demonstrated limited efficacy (2). Current therapies for tauopathies are limited to managing symptoms, as disease modifying interventions against the tau protein have failed in clinical trials(3, 4). A more effective approach may be to target an effector that is known to drive abberant tau phosphorylation that also modulates neuroinflammaoty responses, synergicitlly contributing to disease outcomes.

Dual-specificity tyrosine phosphorylation-regulated kinase 1a (DYRK1A) is one such targetable effector that is dysregulated across multiple neurodegenerative diseases. While expressed in multiple cell types in the brain and periphery, DYRK1A’s highest expression is in neurons and microglia(5). Prior work has shown elevated DYRK1A protein levels in post mortem brain tissue from individuals with primary and secondary tauopathies, including Pick’s disease and AD (6). DYRK1A phosphorylates pathologically-relevant tau epitopes, including threonine 217 (T217), threonine 181 (T181) and serine 396 (S396), promoting the formation of NFTs (7, 8). DYRK1A can also prime other tau-hyperphosphorylating kinases, including glycogen synthase kinase-3β (GSK-3β)(9). Beyond its effects on tau pathogenesis, DYRK1A modulates neuroinflammatory responses, as shown by a dampened inflammatory response following LPS challenge when microglial dyrk1a is reduced(10). Additionally, increased expression of *DYRK1A* contributes to cognitive dysfunction via perturbations in long-term potentiation(11), while *DYRK1A* mutations cause developmental issues and intellectual disability(12). Conversely, Dyrk1a reduction improves performance in behavioral tasks in rodent models of 1) AD, 2) Down syndrome (where Dyrk1a is triplicated and overexpressed), and 3) dyrk1a genetic disorders. (13-15). Collectively, these findings support DYRK1A as a promising therapeutic target for multiple aspects of diverse neurological disorders.

DYRK1A inhibitor development is an active field(16). DYRK1A inhibitors naturally occur in the environment, inspiring development of novel inhibitors such as Leucettine 41 (L41) derived from Leucettamine B, which is produced by the marine sponge *Leucetta microraphis*(17). As DYRK1A belongs to the CMGC family—which includes cyclin-dependent kinases (**C**DKs), mitogen-activate protein kinases (**M**APKs), glycogen synthase kinases (**G**SKs) and CDK-like kinases (**C**LKs)(18)—DYRK1A inhibitors can be potent yet promiscuous compounds, often inhibiting multiple CMGC family kinases or other off-target proteins and leading to adverse long-term outcomes(19, 20). For example, harmine derivatives are potent DYRK1A inhibitors, but their strong affinity towards monoamine oxidase reduces their clinical utility(21). Along these lines, our previously-published DYRK1A inhibitor work with DYR219(22) improved AD-like pathology in the 3xTg-AD mouse model, but required enhancements in half-life, affinity, and selectivity to maximize its therapeutic potential. Thus, we describe a novel small molecule DYRK1A inhibitor, DYR533, which exhibits superior blood-brain barrier (BBB) penetrance, excellent oral bioavailability, and is highly selective within the kinome(15, 23).

We hypothesized that DYRK1A proteins levels would be elevated across multiple primary tauopathies within vulnerable brain regions, and that pharmacological inhibition of DYRK1A with DYR533 would reduce DYRK1A activity and attenuate tau pathology and neuroinflammation in the PS19 model of primary tauopathy. Our findings demonstrate dysregulation of DYRK1A across multiple primary tauopathies and provide evidence that DYR533 mitigates pathological features of tauopathy by preventing DYRK1A autophosphorylation, a process required for kinase activation. Together, these findings support DYRK1A as a potential therapeutic target and highlight DYR533 as a promising multifaceted approach for targeting pathological processes shared across neurodegenerative diseases.

## 2 Methods

### 2.1 Human Tissue

Human postmortem superior frontal gyrus (Sfg) tissue was obtained through the Arizona Study of Neurodegenerative Disorders and Brain and Body Donation Program(24). We used 22 sex-balanced samples of Pick’s disease (n = 4), corticobasal degeneration (CBD; n = 3), and progressive supranuclear palsy (PSP; n = 8), as well as healthy controls (HC; n = 7) to assess DYRK1A protein levels. Pathological assessment of human cases was performed as previously described(24). The average age at autopsy (expired age) was 75.08 years, with a mean post-mortem interval (PMI) of 3.37 hours. We used commercially available enzyme-linked immunosorbent assay (ELISA) kits to quantify DYRK1A protein levels (LSBio, Cat #LS-F8931) in Sfg tissue.

### 2.2 DYR533 and DYRK1A tyrosine autophosphorylation assay

To determine the selectivity of DYR533 a KINOMEscan (*Eurofins*) was performed. This widely-used competitive binding assay screens for kinase inhibitors by assessing the ability of the compound of interest to compete with an immobilized ligand for binding to a DNA-tagged kinase. Quantification was performed using qRT-PCR and reported as an S(35) score. The experimental conditions used in the KinomeProfiler-Eurofins/CEREP radiometric protein kinase assays (a total of 403 kinases) are described in detail at www.eurofinsdiscovery.com/solution/kinase-profiler.

To assess DYRK1A co-translational tyrosine autophosphorylation in the presence of DYR533 and other inhibitors, we conducted an *in vitro* translation assay and quantified phosphotyrosine (pTyr) levels. The NEBExpress^®^ Cell-free E. coli Protein Synthesis System (New England Biolabs, Cat #E5360S) was used to express a DYRK1A construct comprising the kinase domain (residues 126–490) with an N-terminal His tag. Reactions were run in a total volume of 6.25 μL with 40 ng/μL pEXP17-DYRK1A (kind gift of Ulli Rothweiler, Tromsø) at 21°C for 3 h. The DYRK1A inhibitors L41 (Adipogen Cat#MR-C0023)(20), DYR533 and CaNDY(25) were added at 1, 3 and 10 μM concentrations (~ 1% final DMSO concentration) and compared to DMSO control. Tyrosine autophosphorylation of DYRK1A was detected by western blot using a phosphor-HIPK2 (pTyr361) antibody (Thermo Fisher Scientific Cat #PA5-13045, RRID:AB_10987115; 1:500 dilution) as described previously(26). The total amount of the recombinant DYRK1A construct was assessed by detection of the His6-Tag (mouse anti-His antibody, Pharmacia GE Healthcare, Cat #27-4710). Band intensities were quantitated using ImageQuant TL Analysis software (GE Healthcare Life Sciences).

### 2.3 Cell-based DYRK1A degradation assay

The NEBuilder HiFi DNA Assembly Cloning Kit (New England Biolabs, Cat #E5520S) was used to insert rat DYRK1A cDNA into a version of pcDNA5/FRT/TO already equipped with a C-terminal HiBiT sequence(26). The resulting recombinant DYRK1A-HiBiT construct carries a short N-terminal deletion, because a nonsense mutation was inadvertently introduced at codon 51 during the PCR-based cloning procedure. Stable Flp-In T-Rex HEK293 cell lines for doxycycline-inducible expression of DYRK1A-HiBiT were established by Flp recombinase-mediated integration of the pcDNA5/FRT/TO expression vector according to the manufacturer’s instructions (Invitrogen, Cat#V601020). Cells were grown in Dulbecco’s modified Eagle medium/F-12 (DMEM/F-12, Thermo Fisher Scientific, Cat#11330057) supplemented with 10% fetal bovine serum and maintained at 37°C in a humidified 5% CO_2_ atmosphere. For the degradation assays, cells were resuspended in cell culture medium containing 2 μg/mL doxycycline and dispensed into 96-well tissue culture plates (Sarstedt, Cat#83.1835) at a density of 10,000 cells per well in a volume of 90 μL. The following day, compounds were added to the wells (in triplicates) by dispensing 10 μL of 10-fold concentrated working solution to achieve the desired final concentrations of the compounds or the solvent control (0.1% DMSO). After 24 h of treatment, the culture medium was carefully removed and cells were lysed in 100 μL of ice-cold Passive Lysis Buffer (Promega Corporation, Cat#E1941) on an orbital shaker for 30 min. Cell lysates were cleared by centrifugation (20 min at 20,000 x g), and aliquots of 10 μL of the supernatants were used for Nano-Glo HiBiT Lytic Assay (Promega Corporation, Cat#N3030) to determine the concentration of HiBiT-tagged proteins in the soluble fraction. This assay works through the 11-amino-acid HiBiT peptide complementing with LgBiT protein to produce luminescence directly proportional to the amount of the HiBiT tagged DYRK1A protein in a split luciferase assay(27).

### 2.4 Animals

Two-month-old male and female PS19 mice (Jackson Laboratory Strain #008169) and non-transgenic (NonTg) littermate controls were purchased from Jackson Laboratories (n = 16–18/group, balanced for sex). PS19 mice are heterozygous for the human P301S mutation in the microtubule associated protein – tau (MAPT gene, a known genetic cause of early onset FTD-tau in humans(28). All mice were maintained on a 12-hour light/dark cycle at 23°C with *ad libitum* access to food and water and group-housed with four to five mice per cage. All animal procedures were approved by the Institutional Animal Care and Use Committee of Arizona State University (IACUC). Each animal was weighed weekly, beginning at the start of drug regimen (baseline) until euthanasia. Body weight was used to accurately calculate the amount of drug given to each mouse. Animals were monitored daily. Humane endpoints were established and approved by IACUC. Supplementary Table 1 provides a detailed breakdown of the animals and experimental groups included in each study, including information on attrition throughout the study period.

### 2.5 In vivo Dosing

DYR533 was solubilized in a solution of equal parts polyethylene glycol (molecular weight 400; PEG400) and 0.9% sodium chloride (NaCl). The PEG400 and 0.9% NaCl solution (1:1) without DYR533 served as the vehicle (Veh).

Male and female PS19 and NonTg littermates were randomly assigned to one of four dosing regimens starting at four months of age: Veh, 1.0 mg/kg, 2.5 mg/kg, and 5.0 mg/kg of body weight. Daily intraperitoneal (IP) injections were carried out for four months, beginning at early phases of tau pathology in PS19 mice(28). Doses for each study were determined based on preliminary two-week maximum tolerable dose studies (data not shown).

### 2.6 Behavioral testing

#### 2.6.1. Rotarod

PS19 and NonTg littermates underwent three days of Rotarod testing (Rota-Rod Advanced, TSE Systems) at seven and a half months of age to assess motor coordination and endurance(29) as previously described(30). Briefly, each mouse was trained for two days, followed by a probe day, with six time-spaced trials per day. Trials lasted 90 seconds or until the mouse fell off the spinning rod. On training days, the rotation speed of the rod increased by 0.75rpm/s over 20 seconds to a maximum of 15 rpm. On the probe day, the rod accelerated at 1rpm/s from 0 to 60 rpm. The system was connected to computer software (TSE Rotarod, version 5.1.2, TSE Systems) that measured and recorded each mouse's latency to fall. Mice were pseudo-randomly assigned one of five positions on the rod that changed each trial.

#### 2.6.2. Morris Water Maze

To assess hippocampal-dependent spatial learning and memory, PS19 mice and NonTg littermates were tested in the Morris water maze (MWM) as previously described(30). The MWM consists of a one-and-a-half-meter diameter pool filled with 23–24°C water, tinted white with non-toxic paint to obscure the view of the platform. A clear platform (14 cm diameter) was submerged one cm below the water in one of the pool's quadrants. All mice received four 60s trials per day, with a 30s rest period between each trial for five consecutive days. There were prominent spatial cues placed around the testing room. The hidden platform remained in the same quadrant for all trials and mice, while the start location varied pseudo-randomly across all trials. During each trial, mice were required to locate the hidden platform to escape the pool. If mouse failed to reach the hidden platform in 60s, they were gently guided to its location and remained on the platform for 10s. On the sixth day, a probe trial was conducted in which the platform was removed, and each mouse underwent one 60s trial. All trials were recorded with a video camera, and data was analyzed via EthoVisionXT (Noldus Information Technology). Six animals were unable to participate due to hind-end paralysis(28) and were excluded from testing. An additional six animals were excluded from the analysis due to high immobility (n = 5, average % immobile > 50%) or health concerns (n = 1).

### 2.7 Blood Collection and Plasma Extraction

Blood was drawn via the submandibular vein at the end of each study. A total of 150–200 μL of blood was collected into EDTA-lined tubes (K_2_EDTA; BD, Cat #365974) and inverted ten times to ensure anticoagulation, as previously described(31). Tubes were kept on ice for 60–90 minutes, then centrifuged at 455 x g for 30 minutes at 4°C to separate phases. The plasma layer was decanted and frozen at −80°C for future analysis.

### 2.8 Tissue Harvesting and Processing

Mice from the PS19 study were euthanized at eight months of age. Mice were anesthetized with a mixture of ketamine (120 mg/kg) and xylazine (6 mg/kg) prior to transcardial perfusions with cold 1x phosphate-buffered saline (PBS). Brains from the PS19 study mice were extracted and cut along the midline; one hemisphere was fixed in 4% paraformaldehyde in 1x PBS for 48 hours, then changed into 0.02% sodium azide for storage until sectioning, and the remaining hemisphere was dissected to isolate the hippocampus (Hp) and cortex (Ctx) and frozen for protein extraction. Frozen brain samples were homogenized in a tissue protein extraction reagent (TPER, Cat #78510), supplemented with protease (Roches Applied Science, Cat #11836153001) and phosphatase (Millipore, Cat #524625-1SET) inhibitors. Samples were centrifuged for 30 min at 21,130 x g and the supernatant was collected as the soluble fraction for ELISAs and BioRad Luminex assay. 70% formic acid was added to the remaining pellet from PS19 study samples which were homogenized and centrifuged as previously mentioned. The insoluble supernatant was collected and added to a neutralization buffer at a 1:9 ratio to be used as the insoluble fraction for ELISAs.

### 2.9 ELISAs and BioRad Luminex Multi-plex Cytokine Assay

We used commercially-available ELISAs for assessment of homogenates from multiple brain regions. Murine DYRK1A was quantified (LSBio, Cat #LS-F7258) in soluble Hp and Ctx homogenates. We measured human phosphorylated tau (pTau) at threonine 217 (T217; MyBioSource, Cat. #MBS1608795) in the soluble Ctx fraction, and pTau at threonine 181 (T181; Invitrogen-Thermo Fisher, Cat. #KHO0631) and serine 396 (S396; Invitrogen-Thermo Fisher, Cat. #KHB7031) in both soluble and insoluble Hp and Ctx fractions. Because T181 and S396 are established DYRK1A-responsive tau phosphorylation sites and have been shown to be directly phosphorylated by DYRK1A, we prioritized Hp and Ctx tissue for assessment of these sites to evaluate potential effects of DYRK1A inhibition on tau phosphorylation. The remaining Ctx tissue was used to quantify pTau T217 and the expression of pro- and anti-inflammatory cytokines and chemokines. Additionally, we used the Bio-plex^®^-200(32) with Bioplex Manager software (Version 6.2) to quantify the levels of 23 different pro- and anti-inflammatory cytokines and chemokines (Bio-Plex mouse cytokine 23-plex kit; Bio-Rad, Cat #M60009RDPD) in Ctx tissue from PS19 mice as previously described(31). GM-CSF was excluded because values fell outside of the detectable range, thus we report on 22 cytokines. All samples were run in duplicate wells.

### 2.10 Statistical analysis

Analyses were conducted using GraphPad Prism 10.3.0 with significance level set at *p* < 0.05. One-way ANOVA were conducted to assess statistical significance of human DYRK1A. Spearman Rho correlation analyses were performed for human DYRK1A Sfg and pathological measures, including the last Mini-Mental State Examination (MMSE) score, Braak stage, and brain weight. For *in vitro* assays, to assess DYRK1A tyrosine autophosphorylation and degradation, treatment effects were analyzed using repeated measures ANOVA and Dunnett’s multiple comparison test to compare treated samples with the Veh. Weight change, DYRK1A levels, MWM data (latency to platform, speed, distance) and Rotarod training days 1 and 2 were analyzed using two-way ANOVA. Probe day for MWM and Rotarod, pTau, TNF- , and BioPlex cytokine/chemokine quantifications were analyzed using one-way ANOVA. Post hoc corrections were used as recommended by Prism. Bioplex post-hoc testing was performed in GraphPad Prism 10.3.0 with Šidák’s correction for all, except Eotaxin which violated the assumption of normality. For Eotaxin, a Brown-Forsythe ANOVA with Dunnett’s T3 corrections for multiple comparisons was used. Animals were randomly assigned to dose groups and all analyses were conducted blindly to ensure robust and unbiased results. Statistical outliers were identified using the ROUT and Grubbs method. Statistical outputs for all analyses are presented in Supplementary Table 2.

## 3 Results

### 3.1 DYRK1A is elevated across primary tauopathies and correlates with clinical and neuropathological outcomes.

**Figure 1:**
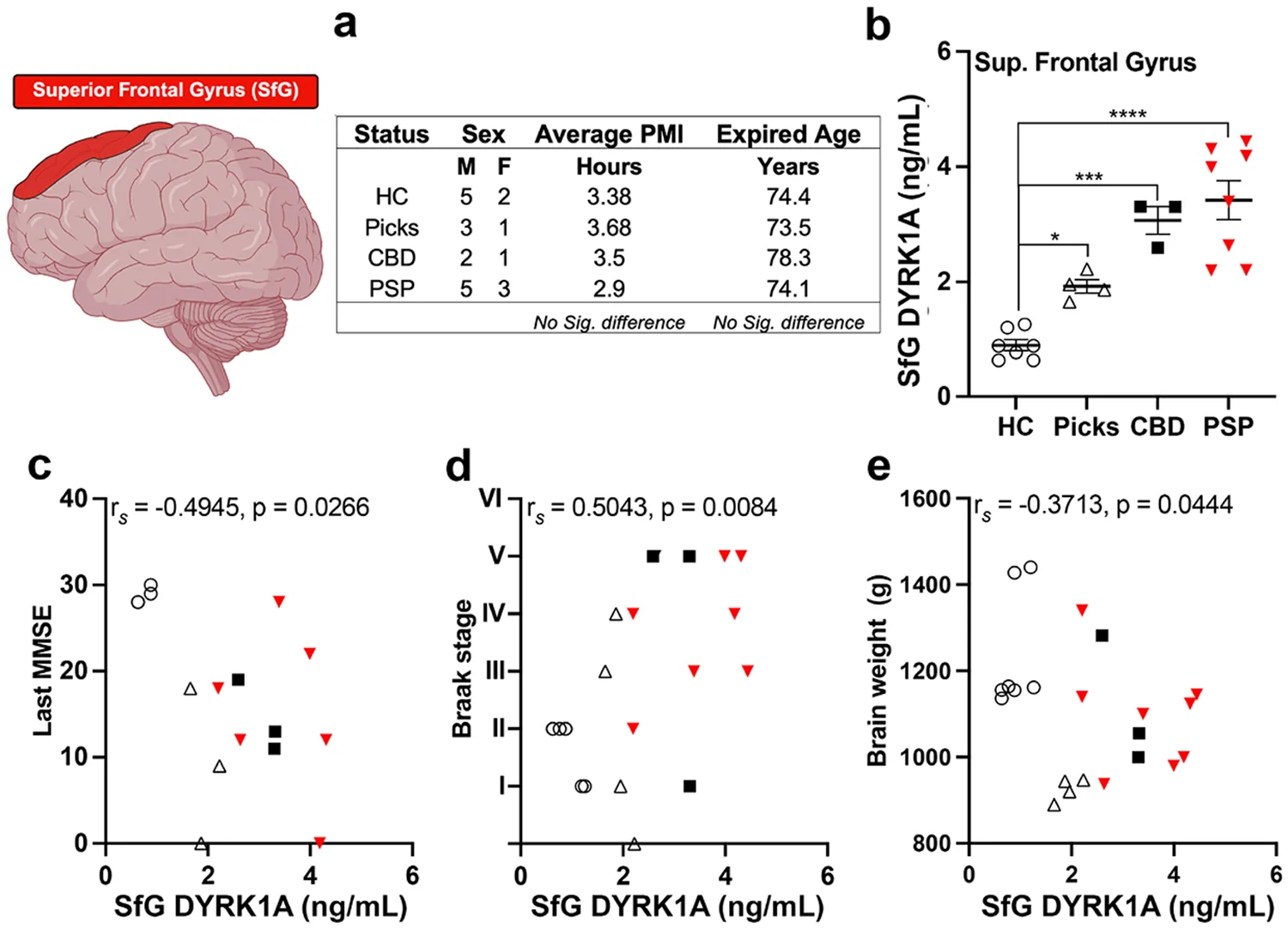
(a) Demographics of tissue used. No significant difference were found in postmortem interval (PMI) nor age at death across the four groups. (b) DYRK1A levels were significantly increased in post-mortem superior frontal gyrus (Sfg) tissue from primary tauopathy cases—including Pick’s disease (n = 4 subjects), corticobasal degeneration (CBD; n = 3 subjects), and progressive supranuclear palsy (PSP; n = 8 subjects) —compared with healthy controls (HC; n = 7 subjects). (c, d, e) Spearman’s rank-correlation analyses revealed that DYRK1A protein levels in the SFG were significantly negatively correlated with Mini-Mental State Examination (MMSE) scores and brain weight, but positively correlated with Braak stage. Data represent mean ± SEM. ns = not significant; *p < 0.05, **p < 0.01, ***p < 0.001, ****p < 0.0001

Previous reports demonstrate DYRK1A elevation in Pick’s disease(6, 33). Here, we quantified DYRK1A protein levels in superior frontal gyrus (Sfg) tissue from individuals with primary tauopathies—including Pick’s, corticobasal degeneration (CBD), and progressive supranuclear palsy (PSP)—and healthy controls (HC; Fig. 1a)(24). Notably, we did not find any significant differences in expired age or PMI across the groups (Fig. 1b). We did find significant elevation of DYRK1A protein levels in all disease cases compared to HC (Fig. 1b; *p* < 0.0001). DYRK1A was strongly negatively correlated to last MMSE(34) (Fig. 1c; *p* = 0.0266), positively correlated with Braak stage(35) (Fig. 1d; *p* = 0.0084), and negatively correlated with brain weight (Fig. 1e; *p* = 0.0444). Our findings demonstrate for the first time that DYRK1A protein levels are elevated in CBD and PSP, and corroborates prior reports of increased DYRK1A in Pick’s disease(6).

### 3.2 Mechanistically, DYR533 inhibits tyrosine autophosphorylation and induces degradation of DYRK1A *in vitro.*

**Figure 2:**
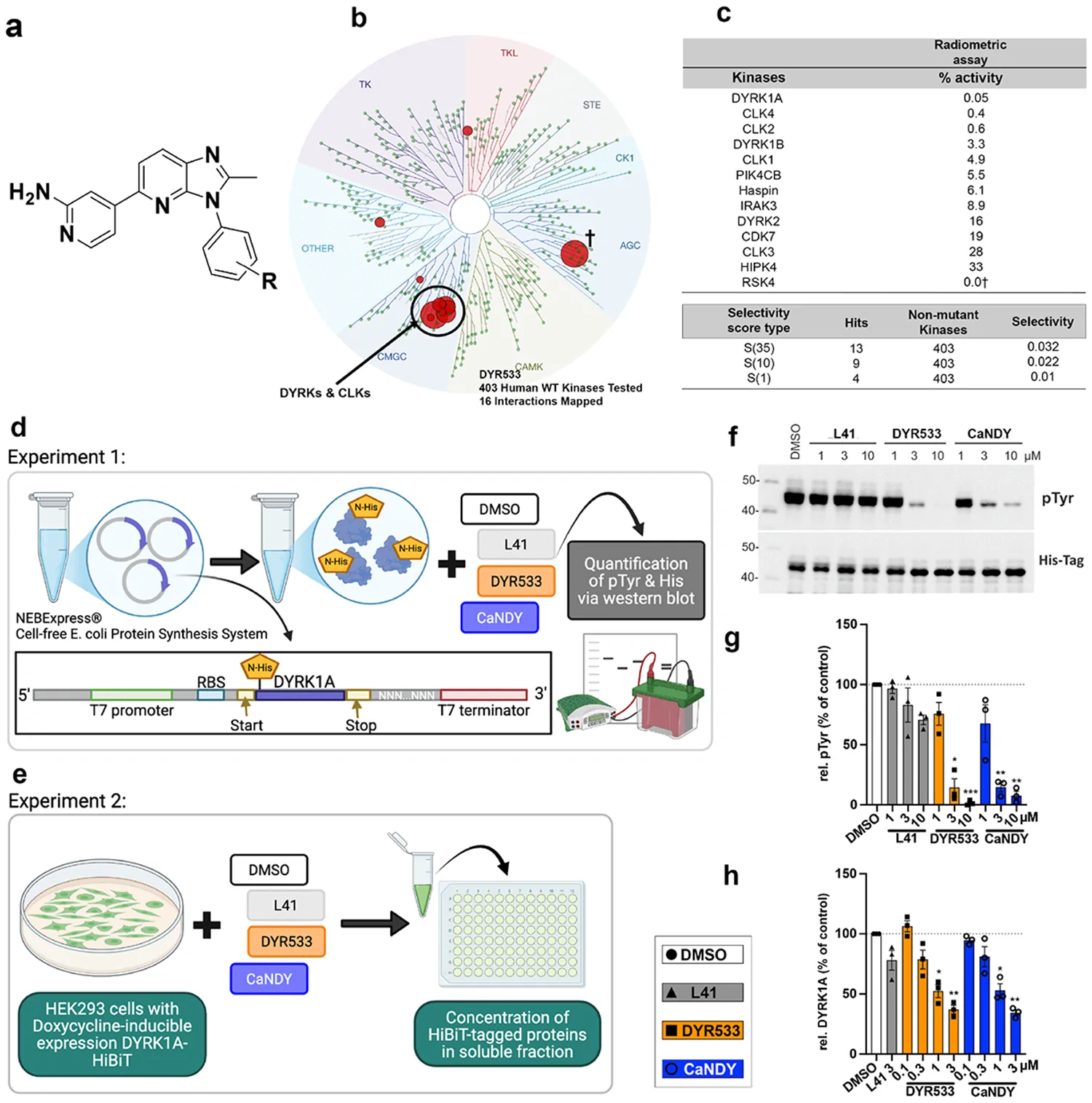
(a) Chemical structure of DYR533. Kinome profiling of DYR533 (1 μM) was conducted across 403 wildtype (WT) human kinases using the Eurofins DiscoverX KinomeScan platform (Online Resource 2). (b) A TREEspot kinase map illustrates binding interactions, with larger circles indicating stronger binding. (c) Selectivity metrics are shown for kinases with percent control <35% ((number of non-mutant kinases with %Ctrl <35)/(number of non-mutant kinases tested)). S-scores represent the fraction of kinases bound at thresholds of <1, <10, and <35, excluding mutant variants. Kinases inhibited >85% are highlighted in bold. Cell-free and *in vitro* assays confirmed that DYR533 blocks DYRK1A intramolecular tyrosine autophosphorylation—similar to the established DYRK1A inhibitor CaNDY—and promotes DYRK1A degradation. (d) Experimental design for phosphotyrosine (pTyr) assays. (e) Western blot results from the pTyr assay and quantification showing reduced pTyr levels following treatment with DYR533 or CaNDY at 3 and 10 μM. (f) DYRK1A degradation assays. (g) DYRK1A degradation assay results demonstrating decreased DYRK1A levels after treatment with 1 or 3 μM DYR533 or CaNDY. Data are presented as mean ± SEM. *p < .05, **p<0.01, ***p < 0.001, ****p < 0.0001

We screened our DYRK1A inhibitor, DYR533 (Fig. 2a) for interactions with 403 human wildtype (WT) kinases (KINOMEscan – *Eurofins*) (Fig. 2b)(36). Larger circles indicate stronger binding, with kinases grouped by family and DYRK and CLK kinases circled within the CMGC family. Results from the kinome scan are summarized via S(35) score, corresponding to the proportion of the total kinases with signal inhibited to 35% of the control value for each kinase(37) (Fig. 2c; Supplementary Table 3). Key kinases with significant S(35) scores selected for follow-up include: DYRK1A (K_d_ 1.4 nM), DYRK1B (K_d_ 11nM), DYRK2 (K_d_ 35 nM) CLK1 (K_d_ 13 nM), CLK2 (K_d_ 59 nM), CLK3 (K_d_ 530nM) and CLK4 (K_d_ 26 nM) (Fig. 2c; Supplementary Table 3). RSK4, a ACG family member, is a false positive confirmed through two subsequent full K_d_ determination (RSK K_d_ > 10 nM, n = 2). Both times the K_d_ was < 10 nM. RSK has not been seen in any other scan of close analogs of DYR533 further supporting it as a false positive.

DYRK1A is autophosphorylated during the folding process at tyrosine 321 (Tyr321)(38), allowing its catalytic domain to acquire a mature conformation and rendering it active towards serine and threonine residues(38). Inhibiting Tyr321 autophosphorylation is thought to inhibit DYRK1A irreversibly, leading to its inactivation and degradation(39). We quantified pTyr in the presence of DYR533 (Fig. 2d) and included the well-characterized DYRK1A inhibitors L41(20, 36) and CaNDY(25) for comparison. DYR533 and CaNDY significantly reduced pTyr compared to untreated control samples (Fig. 2f, g; *p* = 0.0049) at 3 μM (*p* = 0.0228 and *p* = 0.0062, respectively) and 10 μM (*p* = 0.0005 and *p* = 0.0044, respectively). However, no reduction was observed with L41 at any concentration. This suggests DYR533 and CaNDY, but not L41, can inhibit DYRK1A autophosphorylation.

To examine whether DYR533 induces DYRK1A degradation, HEK293 cells stably expressing HiBiT-tagged DYRK1A were incubated with DYRK1A inhibitors at 0.1.-3.0 μM concentrations. After 24 hours, the amount of soluble HiBiT-tagged DYRK1A in the cell lysates was measured by fragment complementation luciferase assay (HiBiT lytic assay; Fig. 2e). DYR533 and CaNDY reduced relative DYRK1A protein levels (Fig. 2h; *p* = 0.0123) compared to DMSO control at 1μM (*p* = 0.0380 and *p* = 0.0411, respectively) and 3μM (*p* = 0.0093 and *p* = 0.0039, respectively). These results highlight the selectivity of DYR533 and its ability to disrupt the autophosphorylation step required for newly-synthesized DYRK1A to become active, leading to degradation of the protein.

### 3.3 DYR533 reduced DYRK1A protein levels while modestly improving behavior outcomes

**Figure 3:**
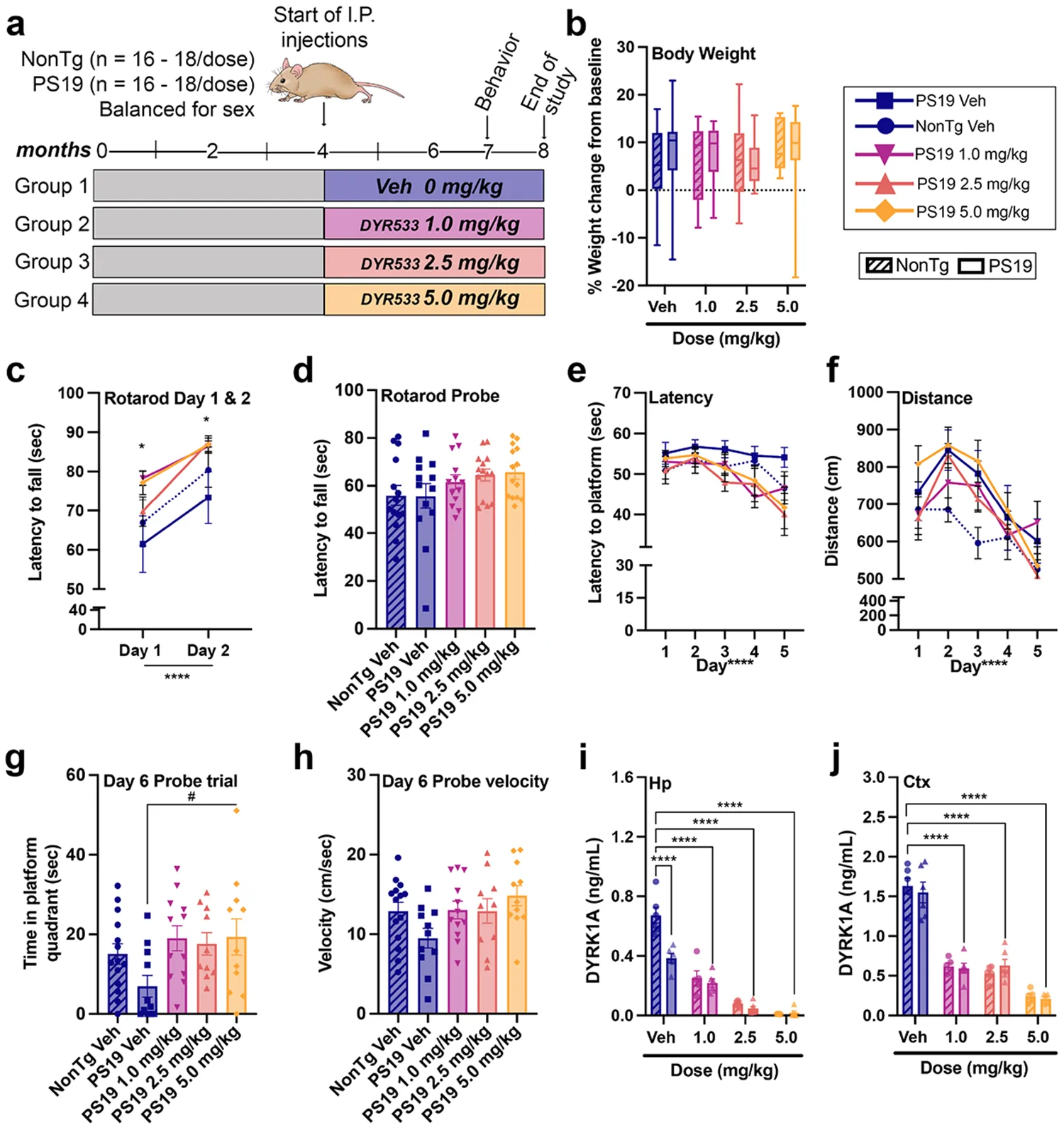
(a) Study timeline and experimental groups. (b) Percent weight change from baseline to study end (n = 9 – 14 mice/group). (c, d) Rotarod performance during training and probe sessions for PS19 groups compared with NonTg Veh (n = 13 – 14 mice/group). (e – h) Morris water maze (MWM) learning and memory performance across treatment groups (n = 7 – 14 mice/group). Quantification of DYRK1A protein levels from (i) hippocampal (Hp) and (j) cortical (Ctx) tissue (n = 5 – 6 mice/group/sex balanced). For box plots, center lines indicate median values; boxes represent the 25th–75th percentiles; whiskers denote minimum and maximum values. Line and bar graphs depict mean ± SEM. *p < 0.05, **p < 0.01, ***p < 0.001, ****p < 0.0001, trend #p = 0.05 < 0.06.

We tested the efficacy of DYR533 in PS19 mice (Fig. 3a). No differences in percent weight change from baseline to end of study were found (Fig. 3b). At 7.5 months of age, mice were tested on a battery of behavioral tasks. Reporting of behavioral task results are focused on performance of all PS19 treated with DYR533 (PSDYR) and PS19 Veh (PSVeh) compared to NonTg Veh, as we did not observe any negative outcomes nor benefits in DYR533-treated NonTg mice. During the two-day Rotarod training period, we found a significant main effect of day (*p* < 0.0001) and group (*p* = 0.0487) for latency to fall (Fig. 3c). A trend toward significantly longer latency to fall was found between the PSDYR 1.0 mg/kg (*p* = 0.071) and 5.0mg/kg (*p* = 0.073) compared to the PSVeh. No significant differences were found in the probe day of the Rotarod (Fig. 3d, p = 0.1753). Next, animals were tested in the Morris water maze (MWM) to assess spatial learning and memory. Six animals were excluded from participation due to hind-end paralysis(28), and an additional six animals were excluded from the analysis due to high immobility (n = 5, average % immobile > 50%) or health concerns (n = 1), which is common in the PS19 mouse strain. During the learning phase, latency to the platform decreased over time (Fig. 3e; *p* < 0.0001). We also analyzed distance (Fig. 3f) and found a significant main effect of day (*p* < 0.0001), but no difference between groups. During the day six probe trial, PSVeh showed a trend towards less time in the correct quadrant compared to PSDYR and NonTg Veh (Fig. 3g, p = 0.0588). Velocity was not significantly different between groups, demonstrating that MWM performance could not be attributed to swim speed differences (Fig. 3h; *p* = 0.0714).

We next quantified DYRK1A protein levels in Hp tissue (Fig. 3i). DYR533 signficantly reduced DYRK1A levels (*p* < 0.0001); a genotype by dose interaction (*p* < 0.0001) and subsequent follow-up revealed lower DYRK1A in PSVeh compared to NonTg Veh mice, and DYR533 significantly reduced DYRK1A in both PS19 and NonTg mice. Similarly, DYR533 significantly reduced DYRK1A levels in the Ctx (Fig. 3j; *p* < 0.0001) in both PS19 and NonTg mice. These findings demonstrate that treatment with DYR533 significantly reduced DYRK1A levels in the Hp and Ctx of PS19 mice, confirming that DYR533 reduces kinase levels in addition to inhibiting its activity.

### 3.4 DYR533 significantly reduced phosphorylated tau at disease-relevant epitopes.

**Figure 4:**
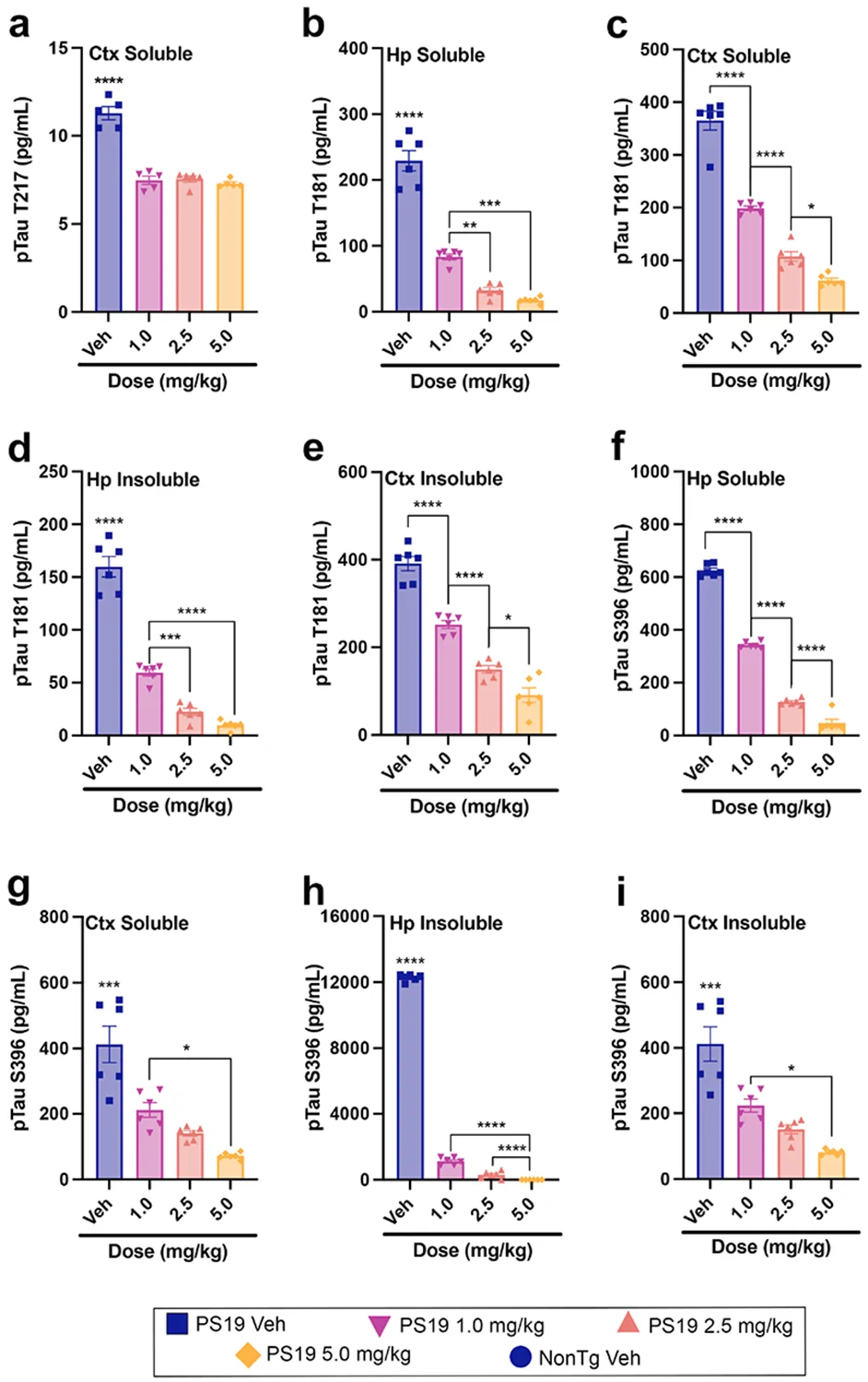
Quantification of (a) pTau T217 in Ctx (n = 5 mice/group); (b – e) pTau T181 in Hp and Ctx (n = 6 mice/group); and (f – i) pTau S396 in Hp and Ctx (n = 6 mice/group/sex balanced). Bar graphs represent mean ± SEM. *p < 0.05, **p < 0.01, ***p < 0.001, ****p < 0.0001.

The P301S mutation of the *MAPT* gene is responsible for the tau pathology observed in PS19 mice, mimicking primary tauopathies. These mice produce human tau that is hyperphosphorylated at disease-relevant epitopes previously mentioned(28). We found that Ctx soluble pTau T217 was significantly reduced (Fig. 4a; *p* < 0.0001) in all PSDYR compared to PSVeh. DYR533 also significantly reduced pTau T181 (p < 0.0001) within soluble (Fig. 4b, c) and insoluble (Fig. 4d, e) tissue fractions of Hp (Fig. 4b, d) and Ctx (Fig. 4c, e), with higher doses of DYR533 resulting in greater T181 reduction. Similarly, DYR533 treatment lowered Hp soluble pTau S396 (Fig. 4f; *p* < 0. 0001) in a dose dependent manner (5.0 (*p* < 0.0001) < 2.5 (*p* < 0.0001) < 1.0 mg/kg (p < 0.0001) < Veh) and Ctx soluble pTau S396 was reduced in PSDYR (Fig. 4g; *p* < 0.0001), with significant differences between high and low doses. Insoluble S396 was also decreased in the Hp (Fig. 4h; *p* < 0.0001) and Ctx (Fig. 4i; *p* < 0.0001) in PSDYR compared to PSVeh, with higher doses showing more significant reductions. Together, these findings demonstrate that DYR533 robustly reduces pathological tau phosphorylation, supporting its therapeutic potential in primary tauopathy.

### 3.5 DYR533 significantly reduced pro- and anti-inflammatory cytokines and chemokines in blood and brain

**Figure 5:**
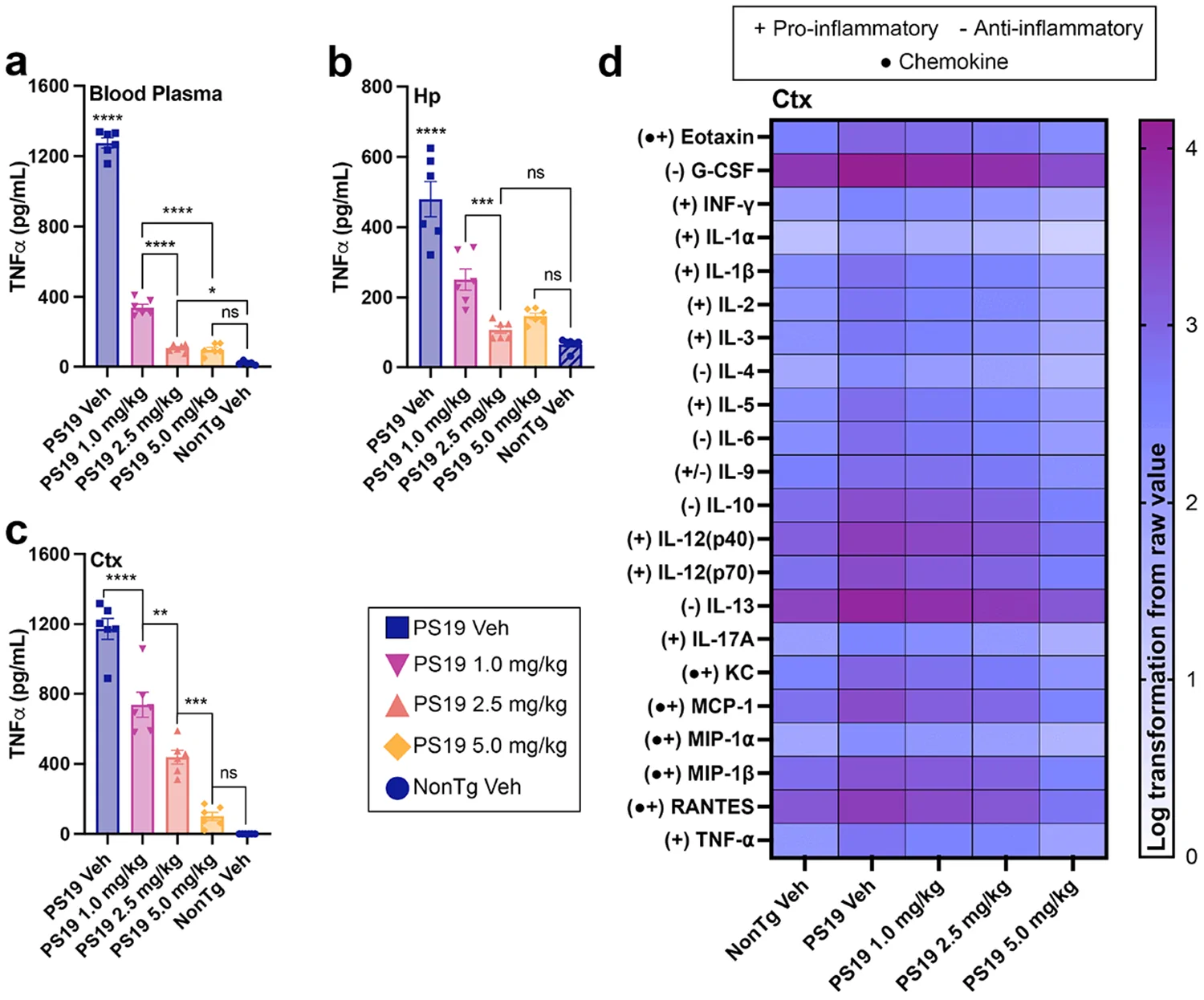
TNFα levels in (j) blood serum (n = 5 – 6 mice/group), (k) Hp (n = 6 mice/group), and (l) Ctx (n = 6 mice/group). (m) Heat map illustrating cytokine and chemokine reductions in Ctx of PS19 mice treated with DYR533, shown as log transformation from raw value (n = 6 mice/group/sex balanced). Bar graphs represent mean ± SEM. *p < 0.05, **p < 0.01, ***p < 0.001, ****p < 0.0001.

We next assessed the impact of DYR533 on tumor necrosis factor (TNF)-α, a pro-inflammatory cytokine elevated across multiple primary tauopathies and implicated in tau pathogenesis(40). We found reductions in peripheral blood plasma (Fig. 5a; *p* < 0.0001) in PSDYR compared to PSVeh. TNF-^ levels after DYR533 treatment followed the same reductions in Hp (Fig. 5b; *p* < 0.0001) and Ctx (Fig. 5c; *p* < 0.0001), with 5.0 mg/kg showing no difference from NonTg (*p* > 0.2). These results show that DYR533 significantly reduced TNF-α in plasma, Hp, and Ctx, with the highest dose rescuing levels to those of NonTg Veh mice. To determine whether DYR533 reduced other inflammatory molecules observed in tauopathies, we next quantified 22 inflammatory molecules (cytokines and chemokines) simultaneously in Ctx homogenates. The 22 quantified inflammatory molecules were significantly elevated in PSVeh compared to NonTg Veh (*p* < 0.0001); comparisons between PSDYR (all doses) and PSVeh showed reductions in 18 out of the 22 measured (Supplementary Table 2, Supplementary Fig. 1 and Fig. 5d; *p* < 0.05), with 5.0 and 2.5 mg/kg doses significantly lowering 22 out of 22 (*p* < 0.05). PSDYR mice dosed at 5.0 and 2.5 mg/kg expressed levels of 21 out of 22 chemokines and cytokines equivalent to NonTg Veh mice, and PSDYR 5.0 mg/kg RANTES levels were significantly lower than NonTg Veh (*p* = 0.0119). Collectively, these findings demonstrate that DYR533 reduces tau hyperphosphorylation and normalizes inflammatory signaling in primary tauopathy further emphasizing its multi-faceted functionality as a cross-disease therapeutic.

## 4 Discussion

We observed increased DYRK1A protein levels in primary tauopathies including Pick’s disease, consistent with previous work(6), CBD and PSP; these elevations were inversely correlated with last MMSE score and brain weight while positively correlated with Braak stage. In PS19 mice, a model of primary tauopathy, baseline DYRK1A protein levels were not elevated in cortices or hippocampi of PS19 mice compared to NonTg littermates. PS19 pathology is driven by mutant human tau, which, on its own, is not known to alter baseline DYRK1A protein expression, rather DYRK1A is known to drive tau hyperphosphorylation(41-43). While DYRK1A is ubiquitously expressed in different cell types, the dosage varies across brain regions(6). Regardless, DYR533 significantly lowered DYRK1A in PS19 and NonTg mice demonstrating its ability to reduce DYRK1A expression *in vivo*. We validated the therapeutic potential of DYR533 in mitigating tau pathology, neuroinflammation, and modestly improving performance in motor and spatial cognitive tasks. These results highlight DYRK1A dysregulation in primary tauopathy and support the use of DYRK1A inhibitors, like DYR533, as therapeutic interventions for a multitude of neurodegenerative diseases.

Mechanistically, DYR533 prevented the autophosphorylation of tyrosine, similarly to CaNDY, effectively rendering the kinase inactive. A common limitation of kinase inhibitors is their promiscuity(44), making the high specificity of DYR533 (S(35) selectivity score of 0.027 at 1μm) particularly desirable compared to CaNDY(23). The next closest kinases inhibited by DYR533 are DYRK1B, CLK1, and CLK4, but at 10x lower affinity. CaNDY inhibits DYRK1B, CLK1, CLK2, and Haspin, which phosphorylates histone H3, by over 90% at 1μm(25). Additionally, as an ATP-competitive inhibitor, DYR533 also blocks the ATP-binding site of DYRK1A preventing kinase function. We demonstrate that DYR533 promotes DYRK1A degradation *in vitro* and observed significant reductions in DYRK1A levels in PS19 mice treated with the drug. DYRK1A degradation has previously been shown via E3 ubiquitin ligase SCF^βTrCP^ in HEK293 cells(45). In 2019, Velazquez, et al.(22) showed that DYR219, a compound similar to DYR533, reduced DYRK1A protein via the proteasome. Our present findings suggest DYR533 may act through a shared cellular protein-disposal mechanism, though future studies are required to confirm.

Tau hyperphosphorylation is a hallmark of various neurodegenerative diseases, yet there are currently no effective therapies that directly target aberrant tau. We demonstrate that DYR533 treatment reduced the phosphorylation of tau at T217, T181 and S396(7, 8), pathologically relevant epitopes in primary tauopathies. However, there are various mechanisms by which DYR533 may have mitigated tau pathology. Previous studies have shown that DYRK1A overexpression increases tau mRNA stability, thereby reducing its degradation and leading to elevated tau expression(46). Tau exists in six isoforms that vary by the number of N-terminal inserts and by the presence of either three or four microtubulebinding repeats (3R or 4R), determined by inclusion or exclusion of exon 10(47). An imbalance in 3R:4R tau isoform expression is a characteristic feature of tauopathies(47-49). DYRK1A overexpression promotes exon 10 exclusion(50), increasing 3R tau levels. Therefore, inhibiting DYRK1A may help preserve exon 10 inclusion and mitigate tau isoform imbalance driving pathogenesis. Additionally, DYRK1A primes other kinases, including GSK-3β, which phosphorylates tau(51), thus DYR533 may reduce pTau levels indirectly by modulating GSK-3β activity. Together, the combined effects of DYRK1A inhibition, reducing tau phosphorylation, preventing exon 10 exclusion, and decreased priming of kinases such as GSK-3β, offer a plausible explanation for the robust reduction in pathological pTau observed here.

An alternative mechanism to explain mitigation of tau hyperphosphorylation with DYR533 treatment was the significant reduction of systemic inflammatory molecules. Although DYRK1A is highly expressed in microglia(5), its specific role in this cell type remains unclear. Some studies have indicated that DYRK1A plays a role in peripheral inflammatory signaling(52) and contributes to immune responses in mouse models of LPS-induced neuroinflammation(10). We observed significant reductions in TNF-α levels in both blood plasma and brain tissue, supporting the potential use of circulating blood TNF-α for monitoring therapeutic response to DYR533 treatment. Moreover, in Ctx homogenates from the PS19 mice, we found a significant reduction in multiple pro- and anti-inflammatory cytokines and chemokines. Neuroinflammation exacerbates tau pathology in neurodegenerative disease(53, 54); for example, interlukin (IL)-1β activates kinases such as p38-mitogen activating protein kinase (MAPK) and GSK-3β(53), which both promote tau phosphorylation and aggregation(55). Blocking IL-1 signaling via IL-1 receptor antagonists can improve cognition and attenuate tau pathology in mouse models of AD and related dementias by reducing the activity of IL-1β-dependent tau kinases, including GSK-3β(56). IL-17 additionally disrupts the BBB, facilitating infiltration of T helper 17 (Th17) cells, which can damage neurons and further increase expression of pro-inflammatory IL-17, −21, and − 22(57). The robust anti-inflammatory effect observed with DYR533 indicates the need to further delineate the role of DYRK1A in neuroinflammation, as it remains unresolved whether these reductions stem from decreased pathological burden, regulation of DYRK1A in microglial altering cytokine production, or other cell-specific functions.

Our findings reveal that DYR533 holds therapeutic potential for neurodegenerative disorders where DYRK1A levels are elevated. In fact, DYRK1A has been implicated in the progression of Parkinson’s disease and amyotrophic lateral sclerosis (ALS) through phosphorylation of ^-syneuclein and TAR DNA-binding protein (TDP)-43, respectively(58, 59), expanding the diagnoses that may benefit from DYR533 treatment. We show that DYR533 treatment lead to reductions in tau pathology and neuroinflammation. While many previously-developed DYRK1A inhibitors have lacked selectivity, contributing to off-target effects and limiting their translational potential, DYR533 demonstrated great potency and selectivity in our preclinical assessments. These findings emphasize that modulation of DYRK1A influences multiple disease-relevant processes. Further work is necessary to elucidate the entirety of underlying mechanisms of DYR533, its affect on other neurodegenerative diseases with mixed-pathology, like Alzheimer’s disease and Down syndrome, and its clinical safety and efficacy.

## Supplementary Material

This is a list of supplementary files associated with this preprint. Click to download.

- SupplementaryTable3Kinome.xlsx
- SupplementaryTable2Stats.xlsx
- SuppFig1PS19.tif
- SupplementaryTable1PS19subjects.docx

Supplementary Fig. 1

(a – v) Individual graphs of cytokine and chemokine levels in PS19 cortex (Ctx) from the multiplex assay shown as a heat map in Fig. 6m (n = 5–6 mice/group/sex balanced). Bar graphs represent mean ± SEM. *p < 0.05, **p < 0.01, ***p < 0.001, ****p < 0.0001

## Data Availability

Data access requests should go through the corresponding author.
